# The use of noninvasive measurements of intracranial pressure in patients with traumatic brain injury: a narrative review

**DOI:** 10.1055/s-0043-1764411

**Published:** 2023-06-28

**Authors:** Bárbara Caroline Dias Faria, Luiz Gustavo Guimarães Sacramento, André Vitor Rocha Queiroz, Fernanda de Andrade Dias Leite, Henrique Lacerda Lage Lopes de Oliveira, Thais Yuki Kimura, Rodrigo Moreira Faleiro

**Affiliations:** 1 Universidade Federal de Minas Gerais, Faculdade de Medicina, Belo Horizonte MG, Brazil.; 2 Faculdade de Ciências Médicas de Minas Gerais, Belo Horizonte MG, Brazil.; 3 Hospital João XXIII, Belo Horizonte MG, Brazil.

**Keywords:** Brain Injuries, Traumatic, Intracranial Pressure, Intracranial Hypertension, Lesões Encefálicas Traumáticas, Pressão Intracraniana, Hipertensão Intracraniana

## Abstract

**Background** The most frequent cause of death in neurosurgical patients is due to the increase in intracranial pressure (ICP); consequently, adequate monitoring of this parameter is extremely important.

**Objectives** In this study, we aimed to analyze the accuracy of noninvasive measurement methods for intracranial hypertension (IH) in patients with traumatic brain injury (TBI).

**Methods** The data were obtained from the PubMed database, using the following terms: *intracranial* *pressure* , *noninvasive* , *monitoring* , *assessment* , and *measurement* . The selected articles date from 1980 to 2021, all of which were observational studies or clinical trials, in English and specifying ICP measurement in TBI. At the end of the selection, 21 articles were included in this review.

**Results** The optic nerve sheath diameter (ONSD), pupillometry, transcranial doppler (TCD), multimodal combination, brain compliance using ICP waveform (ICPW), HeadSense, and Visual flash evoked pressure (FVEP) were analyzed. Pupillometry was not found to correlate with ICP, while HeadSense monitor and the FVEP method appear to have good correlation, but sensitivity and specificity data are not available. The ONSD and TCD methods showed good-to-moderate accuracy on invasive ICP values and potential to detect IH in most studies. Furthermore, multimodal combination may reduce the error possibility related to each technique. Finally, ICPW showed good accuracy to ICP values, but this analysis included TBI and non-TBI patients in the same sample.

**Conclusions** Noninvasive ICP monitoring methods may be used in the near future to guide TBI patients' management.

## Resumo

**Antecedentes** A causa mais frequente de morte em pacientes neurocirúrgicos é devido ao aumento da pressão intracraniana (PIC); consequentemente, o monitoramento adequado desse parâmetro é de extrema importância.

**Objetivos** Avaliar na literatura científica os principais métodos não invasivos de medida da PIC em pacientes com traumatismo cranioencefálico (TCE).

**Métodos** Os dados foram obtidos na base de dados PubMed, utilizando os seguintes termos: *pressão* *intracraniana* , *não invasivo* , *monitoramento* , *avaliação* e *medida* , resultando em 147 artigos. Os artigos selecionados datam de 1980 a 2021, sendo todos estudos observacionais ou ensaios clínicos, em inglês e especificando a medida da pressão intracraniana em traumatismo cranioencefálico. Ao final da seleção, 21 artigos foram incluídos nesta revisão.

**Resultados** Foram analisados os seguintes métodos: diâmetro da bainha do nervo óptico (ONSD), pupilometria, doppler transcraniano (TCD), combinação multimodal, complacência cerebral por meio da análise de ondas intracerebrais (ICPW), HeadSense e visual evocado por flashes de luz (FVEP). A pupilometria não se correlacionou com os valores de PIC, enquanto que o monitor HeadSense e o método FVEP parecem ter uma boa correlação, mas os dados de sensibilidade e especificidade desses métodos não estão disponíveis. Os métodos ONSD e TCD mostraram acurácia de boa a moderada quanto aos valores de IPCi, além de bom potencial para detectar hipertensão intracraniana. Ademais, a combinação multimodal pode reduzir a possibilidade de erro relacionado a cada técnica. Por fim, o ICPW apresentou boa acurácia quanto aos valores de ICPi, mas, no estudo analisado, foram incluídos pacientes com e sem TCE em uma mesma amostra.

**Conclusões** Métodos não invasivos de medição da PIC podem atuar no futuro no manejo de pacientes com TCE como uma potencial ferramenta de triagem para TCE grave e para a detecção de hipertensão intracraniana.

## INTRODUCTION

The most frequent cause of death in neurosurgical patients, especially in severe traumatic brain injury (TBI), 1 is related to the increase in intracranial pressure (ICP). 1 2 It is known that a sustained increase in ICP levels can cause secondary brain injuries, which have potentially fatal consequences for the patient. 1 Hence, adequate monitoring of this parameter is extremely important. 1 2 3 4 5 6 7 8 9 10 11 12 13

Currently, the defined method for monitoring ICP is inserting a catheter into the ventricles of the brain and connecting it to an external pressure transducer. 1 5 However, such an invasive method, in addition to causing complications for the patient, such as infection, hemorrhage and catheter obstruction, is not always available outside of large care centers. 5 Different alternative techniques to the invasive measurement of ICP have been described in the literature; 1 11 however, none have gained space in daily clinical practice, despite the fact that many of them have potential as screening methods for the diagnosis of intracranial hypertension (IH). 7

## Accurate non-invasive methods

In a prior observational study, Robba et al. reviewed the validity of using both optic nerve sheath diameter (ONSD) and transcranial doppler (TCD) as alternatives to invasive ICP (ICPi) measurement among 100 patients. 14 In our study, we not only evaluated a larger number of patients (1,228) from 19 different studies, but also considered other methods not contemplated in this previous study, such as visual flash evoked potential (FVEP), arterial blood pressure (ABP)/flow velocity (FV) ratio, and HeadSense monitoring. In this study, we also present clinical trial data on acoustoelasticity, multimodal combination, and brain compliance using ICP wave morphology not previously addressed in recent reviews. 15

The development of a reliable technique that allows the measurement of ICP in a noninvasive way would make it possible to monitor this parameter in numerous different clinical situations and contexts, in addition to reducing the risk to the patient. We also present results of new methods, such as the HeadSense monitor and brain compliance using ICP waveform. Furthermore, we aim to evaluate in the literature which are the most recognized and studied noninvasive measurement methods for ICP in patients with TBI.

## METHODS

It is a narrative review in which the data were obtained independently by six authors who carried out a search in the PubMed database. The search terms utilized were *intracranial pressure* , *noninvasive* , *monitoring* , *assessment* and *measurement* . The search combination used was: *intracranial* *pressure* [Mesh] AND ( *invasive* OR *noninvasive* ) AND ( *monitoring* OR *assessment* OR *measurement* ). The selection criteria were: i) time frame in 1980 to 2021; ii) observational studies and clinical trials; and iii) studies in which the subject involves the aspects addressed in this review. Also, the following articles were excluded from this review: (i) articles other than the specified inclusion criteria; (ii) articles that were not written in the English language; (iii) articles that did not specify ICP measurement in TBI.

Adopting the aforementioned search descriptors, 147 articles were obtained, and the studies were extracted for abstract screening by 4 authors. Sixty-two articles were found relevant and, therefore, retrieved for full-text read. Disagreements on eligibility were resolved in discussions between the authors who extracted data from studies defined as eligible. After this step, 21 articles were included in this review.

**Figure 1:**
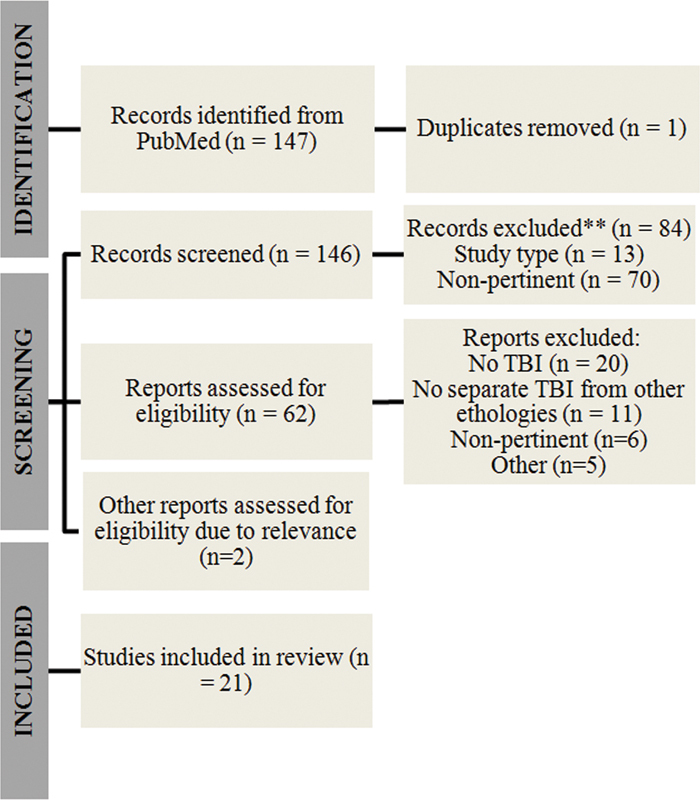
Selection process and identification of studies via databases and registers

In Figure 1 , the details of the selection process are displayed.

### Optic nerve sheath diameter (ONSD)

The ONSD is a new technology of noninvasive means of monitoring ICP, because ICP increases are transduced across the subarachnoid space, increasing the ONSD. 1 2

Raffiz et al. 1 analyzed 41 patients that were divided into 2 groups: traumatic group (TG) and non-traumatic group (NTG). Twenty-one patients (51.22%) were in the TG and 20 patients (48.78%) were in the NTG. The mean age of patients was 33.48 years (19–66); 28 (68.29%) were male and 13 (31.71%) were female. In this study, 75 ocular measurements were performed on all patients, with 39 being measurements (52%) from the TG and 36 measurements (48%) from the NTG. The measurements were repeated on patients with changed ICP values, either elevated or decreased. The control group was composed of 30 patients with presumed normal ICP, and their mean ONSD was 4.57 mm. The study utilized the non-parametric Spearman correlation test, which revealed a significant correlation at the 0.01 level between the ICP and ONSD values, with a correlation coefficient of 0.820. By analyzing the TG and NTG together, a receiver operator characteristic (ROC) curve was generated, and the area under the curve (AUC) had the value of 0.964 with standard error of 0.022 (95% confidence interval [CI] 0.921–1.0). On this ROC curve, the best cutoff point was 5.205 mm with 95.8% sensitive and 80.4% specific in detecting raised ICP. Besides, the overall sensitivity and specificity of 5.47 mm in the TG were higher than in the NTG, 94.4% vs 83.3% and 95.2% vs 93.3%, respectively.

Singer et al. 2 performed a prospective observational study that evaluated the use of noninvasive technologies in screening for TBI, correlating it with ICP. In this study, a total of 244 patients were initially screened, but only 135 patients were enrolled. The patients were divided into 4 groups and the assessment was performed on hospital days 2 and 3. The 1st group was formed by 36 trauma patients with severe TBI, the 2nd group was formed by 39 nontrauma patients, and the 3rd group was formed by 30 trauma patients with mild TBI. Finally, the 4th group included 30 severely injured trauma patients without TBI. On days 2 and 3, the mean right ONSD diameters for severe TBI were, respectively, 5.0 mm and 5.7 mm, compared with 4.7 mm and 4.8 mm for non-TBI trauma and 4.9 mm and 5.1 mm for mild TBI ( *p* < 0.01). Divergently, patients with severe TBI had a mean left ONSD diameter of 5.7 mm and 5.8 mm on postinjury days 2 and 3, compared with 4.8 mm and 4.9 mm for nontrauma patients and 4.8 mm and 5.0 mm diameters for mild TBI ( *p* < 0.01). Specifically on severe TBI patients, the correlation coefficient between right and left ONSD to measurements from an ICPi monitor were 0 ( *p* = 0.07) and 0.996 ( *p* = 0.515), respectively.

In addition, the same correlation on non-TBI right and left ONSD were 0.034 (p = 0.727). and 0.001 ( *p* = 0.996), respectively. Therefore, this study showed that, in spite of ONSD differing significantly bilaterally between non-TBI, mild TBI, and severe TBI on postinjury days 2 and 3, this technology was not found to correlate with ICP.

Maissan et al. 12 evaluated 18 patients admitted to the ICU after TBI using an ultrasound of the optic nerve sheath before, during, and after tracheal manipulation, a situation known to increase ICP. The correlation between ICP and ONSD was analyzed using ROC curve analysis. In all patients, there was an increase during tracheal manipulation of more than 20 mmHg in ICP, associated with a dilation of more than 5.00 mm of the optic nerve sheath. After manipulation, both values returned to their baseline level. A high relationship between ICP and ONSD was found, with R ^2^ = 0.80. At a cutoff point greater than or equal to 5.00 mm ONSD, a sensitivity of 94%, specificity of 98% and an area under the curve of 0.99 (95% CI 0.97–1.00) were found for detection of high ICP.

Strumwasser et al. 13 conducted a prospective blinded study in which trauma patients in need of ICP monitoring underwent an optic nerve sheath ultrasound pre and postplacement of an ICP monitor. Ten patients were analyzed, 114 measurements were obtained and correlated to pre- and post-ONSD with the side of the lesion in the presence of an ICP monitor. Contrary to the study by Maissan et al., 12 this study found, through ROC analysis, that the ONSD does not estimate high ICP well (AUC = 0.36; *p* = 0.01), considering a cutoff point greater or equal to 6.00 mm. The overall sensitivity was 36%, specificity 38%, positive predictive value (PPV) 40%, the negative predictive value (NPV) 16%, and the accuracy for estimating ICP with the ONSD was 37%. A weak correlation was also found between ONSD ICP in unilateral (R ^2^ = 0.45, *p* < 0.01) and bilateral (R ^2^ = 0.21, *p* = 0.01) lesions. The position of the ICP meter did not interfere with ONSD measurements on the right ( *p* = 0.5), on the left ( *p* = 0.4), or right and left side combined ( *p* = 0.3).

Rajajee et al. 4 performed a blinded prospective observational study, which included ICU patients at risk for IH with drains and external ventricular or intraparenchymal ICP monitors. The ONSD was measured simultaneously with the invasive ICP (ICPi) measurement, and a ROC curve was created to determine the optimal ONSD for detection of ICP > 20 mmHg. Sixty-five patients were analyzed and 536 ONSD measurements were performed. Among the diagnoses there were: subarachnoid hemorrhage, TBI, ischemic stroke, intracerebral hemorrhage, and brain tumor. Analysis of curvature ROC (AUC) was 0.98 (95% CI 0.96–0.99; *p* < 0.0001 for AUC = 0.5), and the optimal ONSD for detection of ICP > 20 mmHg was > 4.8 mm with 96% (95% CI 91–99%) and specificity of 94% (92–96%). The sensitivity of the highest cutoff point of ≥ 5.2 mm proposed previously by a few authors was only 67% (58–75%) in this sample, with a specificity of 98% (97–99%).

Soldatos et al. 9 conducted a study with 66 patients, including 58 men, with a mean age of 47 ± 18 years. Fifty of these patients suffered brain injury and 26 were used as controls, without any intracranial pathology. An initial clinical and neuroimaging assessment was performed using the Glasgow coma and the Marshall scales, dividing the patients into those with moderate brain injury (Marshall scale = I and Glasgow coma scale > 8 [n = 18]) and those with severe brain injury (Marshall scale = II–VI and Glasgow coma scale ≤ 8 [n = 32]). Intracranial pressure was measured noninvasively in all patients by TCD simultaneously with ONSD by optic nerve ultrasound. The invasive ICP (ICPi) measurement using an intraparenchymal catheter was performed only in patients with severe brain injury. Patients with severe brain injury had significantly increased ONSD and estimated ICP (eICP) (6.1 ± 0.7 mm and 26.2 ± 8.7 mmHg, respectively; *p* < 0.0001) compared to patients with moderate brain injury (4.2 ± 1.2 mm and 12.0 ± 3.6 mmHg) and control patients (3.6 ± 0.6 mm and 10.3 ± 3.1 mmHg). Furthermore, in patients with severe injuries, there was a very strong correlation between the values of ONSD, eICP (r = 0.80, *p* < 0.0001), and the neuroimaging scale (r = 0.82, *p* < 0.001). In addition, there was a correlation between ONSD values in critically ill patients with the measurement of invasive ICP (ICPi) (r = 0.68, *p* = 0.002), and the best cutoff value of ONSD to predict high ICP was 5.7 mm (sensitivity = 74.1% and specificity = 100%).

The studies of this section are listed in Table 1 .

**Table 1:** Main results of the 21 articles analyzed

Author	Study design	Sample of TBI patients	Noninvasive ICP monitoring method	Summary of findings
Raffiz et al., 2017 1	Prospective observational study	41	ONSD	− ONSD values and ICP: r = 0.820; *p* = 0.01. − Including TBI and non-TBI groups: AUC of 0.964 (CI 95% 0.921–1.0) − Best cutoff point: 5.205 mm with 95.8% sensitivity and 80.4% specificity − Sensitivity and specificity of 5.47 mm in TBI group were higher than in non-TBI group (94.4% vs 83.3% and 95.2% vs 93.3%)
Singer et al., 2021 2	Prospective observational study	135P	ONSD Pupillometry TCD	ONSD − ONSD differs significantly bilaterally between mild TBI, non-TBI, and severe TBI on postinjury days 2 and 3 − ONSD was not found to correlate with ICP. Pupillometry − Significant bilaterally percent changes in pupil diameters ( *p* < 0.01), constriction velocity ( *p* < 0.01) and dilatation velocity ( *p* < 0.01) on postinjury days 1 and 2 − Values obtained from dynamic changes of pupil reliably differentiated severe TBI from mild brain injuries on postinjury days 2 and 3. − Pupillometry values and ICP: *p* > 0.05 TCD − Middle cerebral artery (MCA) peak systolic velocity, MCA flow velocity and common carotid artery flow velocity showed a statistically significant correlation with ICP in severe TBI patients
Maissan et al., 2015 12	Prospective observational study	18	ONSD	− ONSD values and ICP: R2 = 0.80 − Cutoff point ≥ 5.00 mm: sensitivity = 94% and specificity = 98% − AUC 0.99 (0.97–1.00) was found for detection of IH
Strumwasser et al., 2011 13	Prospective observational study	10	ONSD	− ONSD values and ICP in unilateral (R2 = 0.45, *p* < 0.01) and bilateral (R2 = 0.21, p = 0.01) lesions. − AUC = 0.36; *p* = 0.01 − Sensitivity was 36%, specificity 38%, PPV 40%, the NPV 16% and the accuracy for estimating ICP with the ONSD was 37%.
Rajajee et al., 2011 4	Prospective observational study	65	ONSD	− Optimal cutoff point of 4.8 mm: sensitivity = 96% (91–99%) and specificity = 94% (92–96%). − AUC = 0.98 (CI 5 A% 0.96–0.99; *p* < 0.96–0.99; *p* < 0.0001 for AUC = 0.5)
Soldatos et al., 2008 9	Prospective observational study	76	ONSD	− ONSD and ICPi values: r = 0.68; *p* = 0.002 − Best cutoff point: 5.7 mm – sensitivity = 74,1% and specificity = 100% − ONSD and estimated ICP (eICP): o Severe brain injury: 6.1 ± 0.7 mm and 26.2 ± 8.7 mmHg, respectively; *p* < 0.0001 o Moderate brain injury: 4, 2 ± 1.2 mm and 12.0 ± 3.6 mmHg o Control patients: 3.6 ± 0.6 mm and 10.3 ± 3.1 mmHg − Strong correlation between the values of ONSD, eICP (r = 0.80, *p* < 0.0001) and the neuroimaging scale (r = 0.82, *p* < 0.001)
Stevens et al., 2019 10	Prospective observational study	41	Pupillometry	− Pupillometry and ICP: OR 3.36; (95% CI 0.93–13.53; *p* = 0.07) − Decrease in the pupil reactivity may indicate a raised ICP
Cardim et al., 2020 16	Prospective observational study	100	TCD	− ICPtcd and IPCi: r = - 0.17; *p* = 0.097
Rasulo et al., 2017 15	Prospective observational study	20	TCD	− AUC of 0.96 (0.898–1.00), with an estimated best threshold at ICPi of 24.8 mmHg (corresponding a sensitivity of 100% and specificity of 91.2% of the ICPtcd detection of IH)
Robba et al., 2017 6	Prospective observational study	41	ONSD TCD	ONSD − ONSD and ICP: r = 0.76; *p* < 0.05 − AUC of 0.91 (0.88–0.95) to detect cases with IH TCD − Straight sinus FVsv and ICP: r = 0.72; *p* < 0.05 − Combined ONSD and FVsv: r = 0.78; AUC for prediction of ICP 20 mm Hg was 0.93
Cardim et al., 2016 17	Prospective observational study	40	TCD	− ICPtcd pulsatility index: o The best correlation with iICP, including spontaneous changes in ICP > 7 mmHg (R = 0.61) o The best efficacy for ICP dynamics monitoring
Schmidt et al., 2005 7	Prospective observational study	103	TCD	− FV and ABP comparative parameters could infer the ICP with a median absolute difference of 5.7 mmHg when compared to the invasive method.
Ragauskas et al., 20053	Prospective observational study	57	TCD	− The difference in the ICP when comparing the 2 methods was 0.939 mmHg, suggesting the effectiveness of the noninvasive method.
Schmidt et al., 2003 8	Prospective observational study	145	TCD	- Mx and nMx: r = 0.90; *p* < 0.001. - PRx and nPRx: r = 0.62; *p* < 0.001. - The sensitivity of nMx to estimate Mx was 0.92, the specificity of Mx was 0.79 and the fuzzy values were 0.97 and 0.92. - The sensitivity of nPRx to estimate PRx was 0.61 and its specificity was 0.67. - ABP and FV signals cannot be evaluated alone to estimate nICP.
Czosnyka et al., 1998 18	Prospective observational study	96	TCD	- PPV of 94% for the detection of a low CPP (60 mmHg), with r = 0.73 - Estimation error of less than 10 mmHg in 71% of the cases.
Mursch et al., 1995 19	Prospective observational study	28	TCD	- Reductions in diameter were observed: 0.3 to 1.1 mm when there was an increase in ICP. - The relationship between this drop and ICP was not specifically analyzed
Klingelhöfer et al., 1987 20	Prospective observational study	5	TCD	- Changes in the ICP significantly influenced flow patterns - TCD is a useful noninvasive method for gathering information regarding the development of ICP. - The study did not correlate in detail changes in flow patterns and ICP.
Herklots et al., 2017 21	Prospective observational study	14	HeadSense monitor	- HeadSense and ICPi: r = 0.604; *p* < 0.001 - Sensitivity and specificity data were not presented in the study
Zhao et al., 2005 11	Prospective observational study	16	FVEP	- FVEP and ICP: r = 0.97 - No significant difference between the results from noninvasive and ICPi examinations - Sensibility and specificity data were not analyzed in this study
Brasil et al., 2021 26	Prospective observational study	21	Brain compliance using ICPW (B4C monitor)	- B4C sensor measurements and ICP: P2/P1 ratio (r = 0.72) and TTP (r = 0.85) - The B4C P2/P1 ratio threshold of ≥ 1.1 resulted in AUC 0.77 (0.62–0.92), *p* < 0.001, sensitivity 0.88, specificity 0.60) to detect IH
Robba et al., 2020 14	Prospective observational study	30	ONSD PI eICP using TCD NPI using pupillometry	ONSD - AUC of 0.78 (0.62–0.95) to detect IH - Cutoff point of ONSD > 5.3 mm: sensitivity = 67% and specificity = 73% PI - AUC of 0.79 (0.63–0.96) to detect IH - Cutoff point of PI > 1.10: sensitivity = 61% and specificity = 80% eICP using TCD - AUC of 0.83 (CI 0.69–0.98) to detect IH - Cutoff point was eICP > 20 mmHg: sensitivity = 67% and specificity = 87%. NPI - AUC was 0.61 (95% CI 0.49-0.83) to detect IH - Cut-off point of NPI < 4.0: sensitivity = 61% and specificity = 73% COMBINED METHODS - All four methods: AUC 0.91 (0.80–1.00) to detect IH - ONSD with eICP using TCD: AUC 0.92 (0.81–1.00)

Abbreviations: ABP, arterial blood pressure; CPP, cerebral perfusion pressure; CPPn, cerebral perfusion noninvasively; eICP, estimated ICP; FV, flow velocity; FVd, diastolic flow velocity; FVEP, visual flash evoked pressure; FVsd, straight sinus systolic flow velocity; ICP, intracranial pressure; ICPi, standard ICPi; ICPtcd, ICP TCD-derived measurement; IH, intracranial hypertension; ONSD, optic nerve sheath diameter; Mx, autoregulation index invasive; PRx, pressure reactivity index invasive; nMx, autoregulation index noninvasive; nPRx, pressure reactivity index noninvasive; PI, pulsatility index; PPV, positive predictive value; TBI, traumatic brain injury; TCD, transcranial doppler.

### Pupillometry

Pupillometry is another technique that has demonstrated some inverse correlation with ICP. 2 10 In this method, data about both the size and light reactivity of pupils are evaluated, relating them to ICP. 2 10

Singer et al., 2 in their study (mentioned in the ONSD section above), also analyzed the correlation of ICP with pupillometry, which measured absolute values of pupil size and movements over 3.2 seconds, including pupil maximum and minimum size (min. left and min. right), percent constriction (% right, % left), constriction velocity (CV right, CV left), maximum constriction velocity (MCV right, MCV left), dilation velocity (DV right, DV left), and latency for the right and left pupils. In this study, the authors found significant bilaterally percent change in pupil diameter on postinjury days 1 and 2 ( *p* < 0.01), as was CV ( *p* < 0.01) and DV ( *p* < 0.01). Furthermore, the values obtained from dynamic changes of pupil reliably differentiated severe TBI from mild brain injuries on postinjury days 2 and 3. At the same time, there was no correlation between these measurements and ICP in patients with severe TBI.

The study performed by Stevens et al. 10 obtained hourly pupillometry data using a handheld pupillometry device (Neuroptics NPi-100) from 40 patients with TBI. In this group, 31 were male aged between 19 and 79 years. For control, pupillometry readings alone were taken from one control patient with no cranial or neurological trauma. The results of this study showed a weak but statistically insignificant relationship between changes in NPi and ICP (odds ratio [OR] 3.36; 95% CI 0.93–13.53; *p* = 0.07), in which a decrease in the pupil reactivity may indicate a raised ICP.

The studies mentioned in this section are listed in Table 1 .

### Transcranial doppler (TCD)

Transcranial doppler ultrasonography has also been described as a noninvasive ICP measurement method. It detects variations of cerebral blood flow velocity—especially within intracranial arteries in the circle of Willis—by employing a 2 MHz transducer placed on the scalp, possibly correlating with ICP. 5 6

The prospective observational study performed by Singer et al. 2 in 2021 (mentioned in the ONSD section above) also evaluated the TCD method, analyzing if it is possible to differentiate the severity (mild TBI, severe TBI and non-TBI) as well as if the TCD demonstrated a significant correlation with de ICP. Two ultrasound systems were used in the TCD assessment, and both measured the peak systolic velocity (PSV) and end diastolic velocity in the right and left middle cerebral arteries (MCAs), through the transtemporal window and common carotid artery (CCA) at neck midpoint. From these measurements, we calculated the mean flow velocity (MFV), pulsatility index (PI), and ratio of MCA MFV/carotidMFV (Lindegaard ratio). However, neither TCD systems measurements demonstrated consistent or bilateral divergence when comparing severe TBI with mild and moderate TBI. One of them also did not correlate with ICP. In spite of not reliably assessing the differences between non-TBI, mild, and severe TBI, a TCD system showed a statistically significant correlation with ICP in severe TBI by measuring MCA PSV, MCA flow velocity (FV), and CCA FV, which was verified by a *p* < 0.05 for each of these parameters (right MCA PSV, *p* = 0.005; left MCA PSV, *p* = 0.033; right MCA FV, *p* = 0.032 ; left MCA FV, *p* = 0.02; right CCA FV, *p* = 0.018; left CCA FV, *p* = 0.033). Therefore, although they did not differentiate between distinct severities, the TCD measurements showed a significant correlation with ICP. 2

Cardim et al., 16 in 2020, conducted a prospective observational study, aimed to compare the ICP TCD-derived measurement (ICPtcd) with the standard ICPi, using a formula based on the diastolic FV (formula used 12 : CPPn = (MAP x FVd/FVm) + 14; ICPtcd = MAP - CPPn; MAP = mean arterial pressure; FVd = diastolic flow velocity; FVm = mean flow velocity; CPPn = cerebral perfusion noninvasively), which derived from the theoretical idea that ICP increase manifests specific and detectable TCD waveform patterns, including a decrease in the FVd (blood flows more in pulsations and less continuously). It also intended to determine its efficacy to detect IH in TBI patients. In this study, 105 patients were assessed, of which 3 patients did not have an appropriate temporal window and 2 had severe ocular trauma. In this way, 100 TBI patients had a TCD measurement performed on the same day as the insertion of the iICPi monitoring. The study showed that there was no correlation between ICPtcd and ICPi (r =  − 0.17; 95% CI −0.35, 0.03; *p* = 0.097) and, consequently, the TCD-derived FVd is not accurate to estimate and assess ICP noninvasively.

Rasulo et al., 5 in 2017, studied 38 patients with acute brain injury, 20 of whom suffered TBI, 11 aneurysmal subarachnoid hemorrhage, and 7 intracerebral hemorrhage, in a multicenter prospective pilot study that investigated if the TCD eICP accurately detects IH in patients with acute brain injury (12 patients had at least one IH episode) when compared with the gold-standard test ICPi monitoring measurement, using the same formulae as the study conducted by Cardim et al. 16 in 2020, evaluating the MCAs FV. Cerebral blood FV was assessed using TCD sonography. The insonation technique was standard: a low-frequency pulsed 2 MHz ultrasound probe was placed over the acoustic temporal window for insonation of the M1/M2 section of the MCA at a depth ranging from 45 to 55 mm. The MCAs were insonated bilaterally; however, for ICPtcd measurement, the acoustic window ipsilateral to the side of ICP bolt placement was used. For each patient, 3 ICPtcd measurements were performed: the 1st was immediately before placement of ICPi; the 2nd was immediately after insertion of ICPi; and the 3rd was performed between 2 and 3 hours succeeding the second reading—totalizing ultimately 114 ICPtcd readings of which 20 were of elevated ICP. The study considered the cutoff value of 20 mmHg to define IH and found a ICPtcd sensitivity of 100% for this value by analyzing a ROC curve which showed an AUC of 0.96 (95% CI 0.898–1.00), with an estimated best threshold at ICPi of 24.8 mmHg (sensitivity of 100% and specificity of 91.2% of the ICPtcd detection of IH). They also found an estimated conversion between ICPtcd and ICPi of 1.02 (95% CI 0.85–1.36), accounting for a 2% increase in bias for units of ICP ( *p* = 0.59). The results might indicate that ICPtcd, a simple, noninvasive and cost-effective method, might exclude IH accurately in patients with acute brain injury, including TBI cases, in the early phase of hospital admission, but further studies are required to confirm this outcome. 5

Robba et al., 6 in 2017, conducted a prospective single-cohort observational study with 64 patients, attempting to compare distinct ultrasound-based methods accuracy for ICP measurement in patients with severe TBI requiring ICPi monitoring—it evaluated ONSD, venous transcranial doppler (vTCD) and derived characteristics from the straight sinus, including straight sinus systolic flow velocity (FVsv), and arterial transcranial doppler (aTCD) and derived indices (e.g., MCA PI, FVd). The research included 45 TBI cases, and it demonstrated a statistically good correlation between ICPi and OSND (r = 0.76), also ICP and FVsv (r = 0.72). Optic nerve sheath diameter demonstrated the best AUC for differentiating cases with IH from cases without it (AUC = 0.91; 95% CI 0.88–0.95), but the best method was the combination of ONSD and FVsv, which showed the strongest correlation with the ICPi (r = 0.78; AUC for prediction of ICP 20 mm Hg was 0.93) as well as the IH detection accuracy (improvement of AUC values compared with the ONSD alone: 0.93, 95% CI 0.90–0.97, *p* = 0.01 [DeLong test]). Therefore, these findings have shown the combination of ONSD and vTCD as a promising IH screening method and a potential noninvasive ICP measuring method with a quick, low-cost, and simple technology.

Cardim et al., 17 in 2016, performed a prospective cohort study with 40 TBI patients proposing to compare 4 previously described TCD-derived methods with ICPi considering the variability of ICP measurement method based on TCD waveform analysis. The four methods used for IPCtcd estimation were: I) *black-box* model based on interaction between ABP and TCD (applied by Schimdt et al. in 1997—it considered the intracranial compartment as a black-box system, with ABP as the input signal and ICPtcd as the output signal, adjusted by selected hemodynamics parameters); II) based on FVd—inadequate cerebral perfusion derived from decreased cerebral perfusion pressure (CPP) has demonstrated a FVd drop; therefore, it may be an indicator and variable for TCD-measured CPP (based on specific patterns of TCD waveform when there is inadequate cerebral perfusion) ; III) based on critical closing pressure, which denotes a threshold of ABP—below it, microvascular blood pressure is not adequate to prevent cessation and collapse of blood flow—giving information regarding the cerebral hemodynamics; IV) based on ICPtcd pulsatility index (PI), which describes, qualitatively and quantitatively, changes in the TCD waveform morphology derived from cerebral vascular changes; this method relies on the previous observation of an increased PI during rise in ICP. Ultimately, this study showed that method IV had the best correlation with ICPi for recordings including spontaneous changes in ICPi monitoring > 7 mmHg (r = 0.61) and the best efficacy for ICP dynamics monitoring. However, it did not present a satisfactory correlation between ICPtcd variation (difference between maximum and minimum values) with ICPi monitoring variation, which was best demonstrated by method I, considering variations > 7 mmHg (r = 0.68, *p* = 0.06), showing the ability to detect differences in the magnitude of a measured ICP change in time. Method III demonstrated a moderate correlation coefficient (r = 0.35, *p* < 0.05) and congruent 95% CI to method I (9.19 mmHg) yet failed to differentiate normal from raised ICP (AUC = 0.64, *p* > 0.05). Therefore, TCD-based ICP measurement methods may have potential as an initial assessment tool for IH detection in TBI and other conditions, due to its capacity to perceive cerebrovascular derangements caused by ICP changes. The study also showed that ICPtcd methods are potential noninvasive ICP measuring techniques, especially when encompassing a broader set of inputs, such as the evaluated variables (ABP, FV, FVd, and critical closing pressure).

Schmidt et al. 7 described, in 2005, how the fuzzy pattern classification of hemodynamic data could be used to determine ICP. Originally, the authors introduced a method which uses cerebral FV and ABP as parameters to measure the ICP, which did not present great efficiency when used in groups of patients with different clinical parameters, such as arterial CO2 pressure, cerebrovascular state, age, and others. Although, when fitting the patient's heterogeneous data in homogeneous subgroups using fuzzy pattern classification, it was shown that the FV and ABP comparative parameters could infer the ICP with a median absolute difference of 5.7 mmHg when compared to the invasive method.

Ragauskas et al., 3 in 2005, conducted a prospective observational study on 57 TBI patients comparing a new noninvasive method based on a two-depth TCD. This method uses intracranial and extracranial segments of the ophthalmic artery (OA) as pressure sensors and is an accurate indicator of the balance point (Pe = ICP; Pe: external pressure) when parameters of FV waveforms in intra and extracranial OA segments are identical, —with the invasive absolute ICP measurement method. Results show that the difference in the ICP when comparing the 2 methods was 0.939 mmHg, suggesting the effectiveness of the noninvasive method.

Schmidt et al., 8 in 2003, conducted a multicenter study that included Addenbrooke's Hospital, Cambridge, UK (113 patients), and other German medical centers, with a total of 145 TBI (135) and hemorrhagic stroke (10) patients. The objective was to combine methods of continuous autoregulation estimation with assessment of the nICP, to obtain a minimally invasive and continuous assessment. For this, ICPi were monitored, in addition to FV and ABP with TCD. The mean values of ICP, ABP, CPP (CPP = ABP–ICP) and FV were calculated from time integration at 10 second intervals. A moderate-to-high correlation was observed between the invasive and noninvasive estimation of cerebral autoregulation (CA), through the assessment of the autoregulation (Mx) and pressure reactivity (PRx) indices, invasive and noninvasive (nMx and nPRx). The Mx index was calculated as the person correlation coefficient of 36 samples of mean CPP and FV values and describes autoregulation using its definition as a vascular reflex that allows the maintenance of constant cerebral blood flow, regardless of ABP or CPP. The PRx index was calculated as a person correlation coefficient of 36 samples of the mean values of ABP and ICP and, despite being similar to Mx, it takes into account the active regulation of cerebral blood volume. Consequently, if the PRx index is negative, self-regulation is intact (as a correlation coefficient can vary from 1 to -1). That said, TCD characteristics were used to calculate a dynamic transformation formula connecting ICP and ABP. During the analysis, the autoregulatory state was regularly estimated by the correlation between FV and nCPP (nMx) and between ABP and nICP (nPRx). These indices were used to adjust the nICP procedure to the study's CA status. The results showed an important correlation ( *p* < 0.001) between Mx and nMx (r = 0.90) and between PRx and nPRx (r = 0.62). The sensitivity of nMx to estimate Mx was 0.92, the specificity of Mx was 0.79, and the fuzzy values were 0.97 and 0.92. The sensitivity of nPRx to estimate PRx was 0.61, and its specificity was 0.67. The corresponding fuzzy values were 0.77 and 0.78. Therefore, the study concluded that ABP and FV signals cannot be evaluated alone to estimate nICP, because there are changes in the autoregulatory system, such as vasodilation or vasoconstriction, which affect intracranial volume. As a consequence, the assessment of cerebral autoregulation status controlled nICP showed greater precision in estimating the ICP than the corresponding constant procedure. 8

Czosnyka et al. 18 carried out a study in 1998 using TCD, evaluating 96 TBI patients admitted to intensive care with a total of 421 exams. This study aimed to assess the presence of a relationship between CPP and the mean FVd of the MCA. For this, the hemodynamics of the patients, PCI and CBFV were continuously monitored. They showed that the method had a high PPV (94%) for the detection of a low CPP (60 mmHg), with considerable correlation (r = 0.73) and an estimation error of less than 10 mmHg in 71% of the cases.

Mursch et al. 19 described the decrease in the axial diameter of the third ventricle measured with TCD in patients with increased ICP in 1995. The study included 28 people admitted to neurointensive care, victims of TBI. Reductions in diameter were observed, ranging from 0.3 to 1.1 mm when there was an increase in ICP. However, the relationship between this drop and ICP was not specifically analyzed.

In 1987, Klingelhöfer et al. 20 performed an observational study with five patients who developed dissociative brain death despite receiving intensive therapeutic measures. Transcranial investigations of the MCA were recorded using a low frequency (2 MHz) pulsed Doppler device. Flow patterns were recorded either intermittently, or in rapidly progressing courses continuously throughout the course of the disease and stored on magnetic tape. Patients had their ICP measured by an epidural device as well. The study showed that changes in the ICP significantly influenced flow patterns. It was observed that, with continuous increase of the ICP, the mean FV decreased, and the Pourcelote index slightly increased in the TCD. The investigations also showed that ICP and TCD parameters changed according to a progressive reduction in systolic and diastolic FV. A pendular flow was observed with these changes. The results indicate that TCD is a useful noninvasive method for gathering information regarding the development of ICP. However, the study did not correlate in detail changes in flow patterns and ICP.

Regarding ICPtcd measurement methods, studies suggest that this technique is a potential noninvasive ICP assessment, particularly when utilizing a broad set of variables mentioned in this section, such as PI, ABP, FV, FVd, and FVsd. The parameters PI, MFV, and MCA MFV/CCA MFV ratio—calculated by TCD measured PSV and end diastolic velocity in the right and left MCA and CCA, specially in severe TBI patients—demonstrated statistically considerable correlation with ICPi, corroborated by *p* -value < 0.05 for each of these parameters. 2 The PI parameter might also be a potential variable for ICP dynamics monitoring, considering its correlation (r = 0.61) with ICPi measurements recordings, including spontaneous changes in ICPi > 7 mmHg. 17 The combination of ONSD and the parameter FVsd measured with venous TCD demonstrated significant correlation with ICPi (r = 0.78) along with a notable accuracy to detect IH 6 (AUC of 0.93, 95% CI 0.90–0.97; *p* = 0.01). The black-box model, 17 by evaluating the relation between ABP parameter and ICPtcd, had the best correlation with the ICPi variation, considering variation > 7 mmHg (r = 0.68, *p* = 0.06), presenting a potential ability to detect oscillations of the measured ICP changes in time, might be a useful method to assess the ICP during the patient's hospitalization.

When only using TCD-measured FVd to estimate ICP (formulae: CPPn = (MAP x FVd/FVm) + 14; ICPtcd = MAP - CPPn), there was no correlation between ICPtcd and ICPi, with r =  − 0.17. It was also verified that the ABP and FV parameters obtained by TCD cannot be evaluated alone in order to estimate ICPtcd, due to changes in the cerebral autoregulatory system affecting intracranial volume. However, using the same formulae above, ICPtcd measured by FVd demonstrated a sensitivity and specificity to detect IH - ICP between 20 and 24.8 mm - of 100% and 91.2%, respectively. The relationship between CPP and the mean FVd of the MCA in the study by Czosnyka et al. 20 was also described as a promising parameter to estimate ICP, with a high positive value of 94% for detection of low CPP (60 mmHg) and expressive correlation (r = 0.73). Considering these conflicting findings, other studies are important to evaluate the FVd and FVm accuracy to estimate ICP noninvasively. Still, these parameters might be a potential method to detect low CPP and to verify IH.

The studies of this section are listed in Table 1 .

### HeadSense monitor

Herklots et al. 21 performed a prospective comparative clinical trial with 14 severe TBI patients, in which ICPi values were compared to HeadSense HS-100 nICP monitor measures. This nICP monitor device is based on an advanced acoustic signal that is transmitted from one patient's ear to another. Then, this acoustic signal is analyzed through algorithms, and the ICP value is calculated according to the measured energy of the test signal. In this study, 13 (92.9%) patients were male, the mean age was 47 years (range 19–71), and all patients had a sustained TBI and a survival expectancy greater than 72 hours. The exclusion criteria of this study were ear infection, pregnant or lactating women, and CSF or blood leakage in the ear canal. The mean difference between the ICPi and nICP values was 0.5 ± 3.9 mmHg, 18.8% and 38.3% of total measurements had a difference > 5 mmHg and > 3 mmHg, respectively. The Pearson r correlation of the measurement was 0.604 ( *p* < 0.001), but sensitivity and specificity data were not presented in the study.

The study of this section is listed in Table 1 .

### Visual flash evoked pressure (FVEP)

The FVEP shows the integrity of the visual pathway from the retina to the occipital lobe. Increases in ICP induce ischemia, with a consequent reduction in CSF pH and nerve conduction block. This block prolongs the latency period of peak FVEP, and, in this sense, there is a direct correlation between FVEP and ICP levels. 11

In that regard, Zhao et al. 11 described the efficiency of the noninvasive ICP Monitoring System NIP-200 (Chongqing Sunkingdom Medical Instrument Co.,Ltd., Jiulogpo District, Chongqing, China), a method based on the N2 wave response to FVEP. The prospective observational study comparing measurements of ICP using invasive methods and the FVEP in 152 patients (16 of which had brain trauma) showed a linear correlation between FVEP and the invasive methods, with a correlation coefficient (r) of 0.97. Furthermore, this study also showed that there is no significant difference between the results from noninvasive and ICPi examinations ( *p* > 0.05) and showed an average relative error of 13.22%, indicating FVEP as a good option for noninvasive ICP measuring, especially when considered its convenience for bedside use. However, sensibility and specificity data were not analyzed in this study.

The study of this section is listed in Table 1 .

### Brain compliance using ICP waveform

The analysis of brain compliance through intracranial pressure waveform (ICPW) on invasive techniques is a previously described method and evaluates the occurrence of an alteration in CSF hydrodynamics, 22 including arterial pulsation (P1) and cerebral venous flow (P2). Recently, a new noninvasive mechanical sensor (B4C) that detects micrometric cranial deformations was developed, therefore acquiring ICPW in real time monitoring. This promising method demonstrated linear correlation between ICP and skull deformations in experimental models in vitro 23 and in animal testing. 24 25

Brasil et al., 26 in an observational prospective study, compared ICPW parameters (P1 and P2 amplitudes, P2/P1 ratio, and time to peak (TTP) interval) obtained through invasive monitoring with the waveforms obtained by the B4C sensor. This study included 41 patients that suffered traumatic and nontraumatic (subarachnoid hemorrhage, stroke, and tumor) acute brain injury. The sample mean age was 37.6 ± 28.2 years, 22 (53%) of the participants were male, 21 (51%) suffered TBI, and the patients were divided in 3 subgroups: intact skull bone (group A – n = 12 [29%]), large skull fractures or craniotomies (group B – n = 20 [48%]), and craniectomies (group C – n = 9 [21%]). The B4C sensor was positioned 3 cm over the first third of the orbitomeatal line in the frontotemporal region and the data was obtained through a 10-minute session for each patient. Furthermore, a guided manual internal jugular vein (IVJ) compression was performed for 60 seconds at minute 7 to evaluate alterations on ICP values.

In this study, the Bland-Altman's plot indicated agreement between the ICPW parameters obtained using both techniques, and group A showed the highest linear correlation between B4C sensor measurements and invasive measurements of P2/P1 ratio (r = 0.72) and TTP (r = 0.85). Through IVJ compression, 36% of patients overpassed the 20 mmHg ICP cutoff, thus the ROC curve for P2/P1 ratio cutoffs for ICP values > 20 mmHg was calculated. The B4C P2/P1 ratio threshold of ≥ 1.1 resulted in AUC 0.77 (95% CI 0.62–0.92, *p* < 0.001, sensitivity 0.88, specificity 0.60), with better results in group A (AUC 0.90, P2/P1 ratio threshold ≥ 1.2, *p* < 0.001) when compared to group B (AUC 0.78, P2/P1 ratio threshold ≥ 1.1) or group C (AUC 0.70, P2/P1 ratio threshold ≥ 1.1). However, there was no statistically significant difference between these groups.

The study of this section is listed in Table 1 .

### Multimodality

Robba et al. 14 prospectively observed 100 patients that underwent ICPi monitoring due to TBI, subarachnoid hemorrhage, or intracerebral hemorrhage over January 2017 to September 2018. In this sample, 30 (30%) patients were victims of TBI, and the authors analyzed the following indices: ONSD, PI, eICP using TCD, and the NPI measured using automated pupillometry.

To predict IH in TBI patients, the AUC was 0.78 (95% CI 0.62–0.95) for ONSD, obtaining 67% sensitivity and 73% specificity when the cutoff point was ONSD > 5.3 mm. For PI, the AUC was 0.79 (95% CI 0.63–0.96) and PI > 1.10 obtained 61% sensitivity and 80% specificity. In addition, the AUC was 0.83 (95% CI 0.69–0.98) for eICP, demonstrating sensitivity of 67% and specificity of 87% when the cutoff point was eICP > 20 mmHg. Finally, the AUC was 0.61 (95% CI 0.49–0.83) for NPI and NPI < 4.0 had 61% sensitivity and 73% specificity. 14

Using the 4 noninvasive methods combined, the study obtained an AUC 0.91 (0.80–1.00) to detect IH; however, the best AUC 0.92 (0.81–1.00) was obtained with the combination of ONSD with eICP using TCD. 14

The study of this section is listed in Table 1 .

## DISCUSSION

In this narrative review, we included different forms of noninvasive methods for estimating ICP in TBI patients. It is known that the increase in ICP, especially in TBI, is a severe complication, and its adequate monitoring is extremely important. 1 2 3 4 5 6 7 8 9 10 11 12 13 The invasive catheter method is the most utilized, but the indications for invasive ICPi monitoring remain controversial in some brain conditions and also increase risk of complications to the patient. 5 14 In this regard, the use of non-invasive methods might be helpful in these cases, even more in low-income countries with few medical resources.

The ONSD is a new technology of noninvasive means of monitoring ICP. Each study considered a different ONSD cutoff point for high ICP (> 20 mmHg) to determine the sensitivity and specificity for the screening purpose. The [sensitivity/specificity] considering a ONSD cutoff point of 4.8 mm, 5.0 mm, and 5.2 mm were, respectively, [96%/94%], [94%/98%], and [67%/98%]. Thus, the ONSD is a promising method to detect a high ICP noninvasively and should prefer a higher sensitivity or specificity for its screening function with a 4.8-mm or 5.0-mm cutoff, respectively. Furthermore, the results of this review showed that, ultimately, with the exception of the Strumwasser study, 13 most of the aforementioned studies 1 2 4 9 12 demonstrated that the ONSD method has a good accuracy on detecting IH; therefore, it might be a potential screening instrument in neurocritical care.

Also, we found that pupillometry was able to distinguish between severe TBI, mild TBI, and non TBI patients in postinjury days 2 and 3. 2 However, the method did not present a significant correlation between pupillary function and ICP. 2 10 14 Therefore, this technique still cannot replace ICPi measurement devices, even when patients are far from large centers. In the future, after larger studies are conducted in this specific topic, pupillometry could be used as a screening tool for severe TBI in places with few medical resources or in military environments, serving as a supplement to the initial assessment.

The TCD measurement methods are potentially useful noninvasive ICP-assessing techniques, 5 27 notably when encompassing a broad set of variables such as ABP, FV, FVd, and critical closing pressure, demonstrating a significant accuracy in some studies when compared to ICPi. 5 6 8 17 Albeit the method did not differentiate between distinct TBI severities, the ICPtcd measurements demonstrated a potential supplementary function to detect IH in a noninvasive, simple, and cost-effective way. 5 27

The simultaneous use of 4 methods described in this review (ONSD, PI, eICP using TCD, and the NPI measured using automated pupillometry) showed good accuracy to detect IH and the best AUC 0.92 (0.81–1.00) was obtained with the combination of ONSD with eICP using TCD. 14 These findings demonstrate that, even though some indices alone have low capacity to detect IH, the simultaneous combination of two or more methods may reduce the error possibility related to each technique and, therefore, increase accuracy.

Moreover, the noninvasive analysis of ICP waveform morphology, specifically P2/P1 ratio and TTP, through B4C monitor demonstrated good correlation to ICPi values, and the B4C P2/P1 ratio threshold of ≥ 1.1 resulted in AUC 0.77 (0.62–0.92, *p* < 0.001, sensitivity 0.88, specificity 0.60) in their sample. This clinical study corroborates previous findings reported in experimental models in vitro 23 and in experimental animal studies. 24 25 Despite the good and promising results presented, the statistical analysis was performed including the entire sample (TBI and non-TBI patients); therefore, it is not possible to reach a conclusion about its specific use in TBI patients, which was the scope of our narrative review. Nevertheless, the B4C monitor showed good results and further studies specifically with trauma patients will possibly corroborate these findings.

Other methods, such as FVEP and HeadSense monitoring, are convenient for bedside use and showed good correlation to ICP measurements, but sensibility and specificity data were not analyzed in the studies. Therefore, these methods still cannot be reliably used for monitoring ICP until further studies demonstrate that the technology is an accurate procedure to estimate ICP in TBI patients 7 11 22 (the same holds true for two-depth TCD 3 ). However, a huge limitation of the HeadSense nICP monitor in TBI patients is the fact that blood or CSF in the ears interfere with the measurement process; therefore, some patients with skull base fracture might not be monitored with this method.

This narrative review also presents some limitations. We performed our data extraction in only one database, which increases the risk of bias. Furthermore, some of the conflicting findings in these studies may be related to the use of different methodologies, small sample size, sample heterogeneity in the studies, absence of a standard protocol to be used in each method, interobserver variability, and different expertise of operators.

In conclusion, noninvasive ICP monitoring methods may be used in the near future to guide TBI patients management, particularly in low-income countries with few medical resources or if there are controversies on the indication of F monitoring. In this regard, multimodal combination may reduce the error possibility related to each technique.

## References

[1] Raffiz M Abdullah J M Optic nerve sheath diameter measurement: a means of detecting raised ICP in adult traumatic and non-traumatic neurosurgical patients Am J Emerg Med 2017 35 01 150 153 27852525 10.1016/j.ajem.2016.09.044

[2] Singer K E Wallen T E Jalbert T Efficacy of Noninvasive Technologies in Triaging Traumatic Brain Injury and Correlating With Intracranial Pressure: A Prospective Study J Surg Res 2021 262 27 37 33540153 10.1016/j.jss.2020.12.042

[3] Ragauskas A Daubaris G Dziugys A Azelis V Gedrimas V Innovative non-invasive method for absolute intracranial pressure measurement without calibration Acta Neurochir Suppl (Wien) 2005 95 357 361 10.1007/3-211-32318-x_73 16463881

[4] Rajajee V Vanaman M Fletcher J J Jacobs T L Optic nerve ultrasound for the detection of raised intracranial pressure Neurocrit Care 2011 15 03 506 515 21769456 10.1007/s12028-011-9606-8

[5] Rasulo F A Bertuetti R Robba C The accuracy of transcranial Doppler in excluding intracranial hypertension following acute brain injury: a multicenter prospective pilot study Crit Care 2017 21 01 44 28241847 10.1186/s13054-017-1632-2 PMC5329967

[6] Robba C Cardim D Tajsic T Ultrasound non-invasive measurement of intracranial pressure in neurointensive care: A prospective observational study PLoS Med 2017 14 07 e1002356 28742869 10.1371/journal.pmed.1002356 PMC5526499

[7] Schmidt B Bocklisch S F Pässler M Czosnyka M Schwarze J J Klingelhöfer J Fuzzy pattern classification of hemodynamic data can be used to determine noninvasive intracranial pressure Acta Neurochir Suppl (Wien) 2005 95 345 349 10.1007/3-211-32318-x_71 16463879

[8] Schmidt B Czosnyka M Raabe A Adaptive noninvasive assessment of intracranial pressure and cerebral autoregulation Stroke 2003 34 01 84 89 12511755 10.1161/01.str.0000047849.01376.ae

[9] Soldatos T Karakitsos D Chatzimichail K Papathanasiou M Gouliamos A Karabinis A Optic nerve sonography in the diagnostic evaluation of adult brain injury Crit Care 2008 12 03 R67 18477382 10.1186/cc6897 PMC2481450

[10] Stevens A R Su Z Toman E Belli A Davies D Optical pupillometry in traumatic brain injury: neurological pupil index and its relationship with intracranial pressure through significant event analysis Brain Inj 2019 33 08 1032 1038 31021683 10.1080/02699052.2019.1605621

[11] Zhao Y L Zhou J Y Zhu G H Clinical experience with the noninvasive ICP monitoring system Acta Neurochir Suppl (Wien) 2005 95 351 355 10.1007/3-211-32318-x_72 16463880

[12] Maissan I M Dirven P J Haitsma I K Hoeks S E Gommers D Stolker R J Ultrasonographic measured optic nerve sheath diameter as an accurate and quick monitor for changes in intracranial pressure J Neurosurg 2015 123 03 743 747 25955869 10.3171/2014.10.JNS141197

[13] Strumwasser A Kwan R O Yeung L Sonographic optic nerve sheath diameter as an estimate of intracranial pressure in adult trauma J Surg Res 2011 170 02 265 271 21550065 10.1016/j.jss.2011.03.009

[14] Robba C Pozzebon S Moro B Vincent J L Creteur J Taccone F S Multimodal non-invasive assessment of intracranial hypertension: an observational study Crit Care 2020 24 01 379 32591024 10.1186/s13054-020-03105-z PMC7318399

[15] Moraes F M Silva G S Noninvasive intracranial pressure monitoring methods: a critical review Arq Neuropsiquiatr 2021 79 05 437 446[Accessed 20 September 2022], pp. 437–446. Wu J, He W, Chen W, Zhu L. Research on simulation and experiment of noninvasive intracranial pressure monitoring based on acoustoelasticity effects. Med Devices (Auckl). 2013;6:123–131 24009433 10.2147/MDER.S47725 PMC3758219

[16] Cardim D Robba C Czosnyka M Noninvasive Intracranial Pressure Estimation With Transcranial Doppler: A Prospective Observational Study J Neurosurg Anesthesiol 2020 32 04 349 353 31306262 10.1097/ANA.0000000000000622

[17] Cardim D Robba C Donnelly J Prospective Study on Noninvasive Assessment of Intracranial Pressure in Traumatic Brain-Injured Patients: Comparison of Four Methods J Neurotrauma 2016 33 08 792 802 26414916 10.1089/neu.2015.4134 PMC4841086

[18] Czosnyka M Matta B F Smielewski P Kirkpatrick P J Pickard J D Cerebral perfusion pressure in head-injured patients: a noninvasive assessment using transcranial Doppler ultrasonography J Neurosurg 1998 88 05 802 808 9576246 10.3171/jns.1998.88.5.0802

[19] Mursch K Vogelsang J P Zimmerer B Ludwig H C Behnke J Markakis E Bedside measurement of the third ventricle's diameter during episodes of arising intracranial pressure after head trauma. Using transcranial real-time sonography for a non-invasive examination of intracranial compensation mechanisms Acta Neurochir (Wien) 1995 1371-2, discussion 23–2419 23 8748862 10.1007/BF02188774

[20] Klingelhöfer J Conrad B Benecke R Sander D Intracranial flow patterns at increasing intracranial pressure Klin Wochenschr 1987 65 12 542 545 2957547 10.1007/BF01727619

[21] Herklots M W Moudrous W Oldenbeuving A Prospective Evaluation of Noninvasive HeadSense Intracranial Pressure Monitor in Traumatic Brain Injury Patients Undergoing Invasive Intracranial Pressure Monitoring World Neurosurg 2017 106 557 562 28712896 10.1016/j.wneu.2017.07.022

[22] Nucci C G De Bonis P Mangiola A Intracranial pressure wave morphological classification: automated analysis and clinical validation Acta Neurochir (Wien) 2016 158 03 581 588, discussion 588 26743919 10.1007/s00701-015-2672-5

[23] Mascarenhas S Vilela G H Carlotti C The new ICP minimally invasive method shows that the Monro-Kellie doctrine is not valid Acta Neurochir Suppl (Wien) 2012 114 117 120 10.1007/978-3-7091-0956-4_21 22327675

[24] Cabella B Vilela G H Mascarenhas S Validation of a New Noninvasive Intracranial Pressure Monitoring Method by Direct Comparison with an Invasive Technique Acta Neurochir Suppl (Wien) 2016 122 93 96 10.1007/978-3-319-22533-3_18 27165884

[25] Frigieri G Andrade R AP Wang C C Analysis of a Minimally Invasive Intracranial Pressure Signals During Infusion at the Subarachnoid Spinal Space of Pigs Acta Neurochir Suppl (Wien) 2018 126 75 77 10.1007/978-3-319-65798-1_16 29492536

[26] Brasil S Solla D JF Nogueira R C Teixeira M J Malbouisson L MS Paiva W DS A Novel Noninvasive Technique for Intracranial Pressure Waveform Monitoring in Critical Care J Pers Med 2021 11 12 1302 34945774 10.3390/jpm11121302 PMC8707681

[27] Wu J He W Chen W M Zhu L Research on simulation and experiment of noninvasive intracranial pressure monitoring based on acoustoelasticity effects Med Devices (Auckl) 2013 6 123 131 24009433 10.2147/MDER.S47725 PMC3758219

